# Supplementary material for: The interactome of intact mitochondria by cross-linking mass spectrometry provides evidence for coexisting respiratory supercomplexes
Source: Mol Cell Proteomics. 2017 Dec 8;17(2):216–32. doi: 10.1074/mcp.RA117.000470 (PMC5795388; doi:10.1074/mcp.RA117.000470)
Supplement: Supplemental Data [file supp_RA117.000470_133922_0_supp_23978_fzffwf.zip › spectra_annotation/mito_DR_spectra_annotation/350-1-3-1-8-1.pdf]

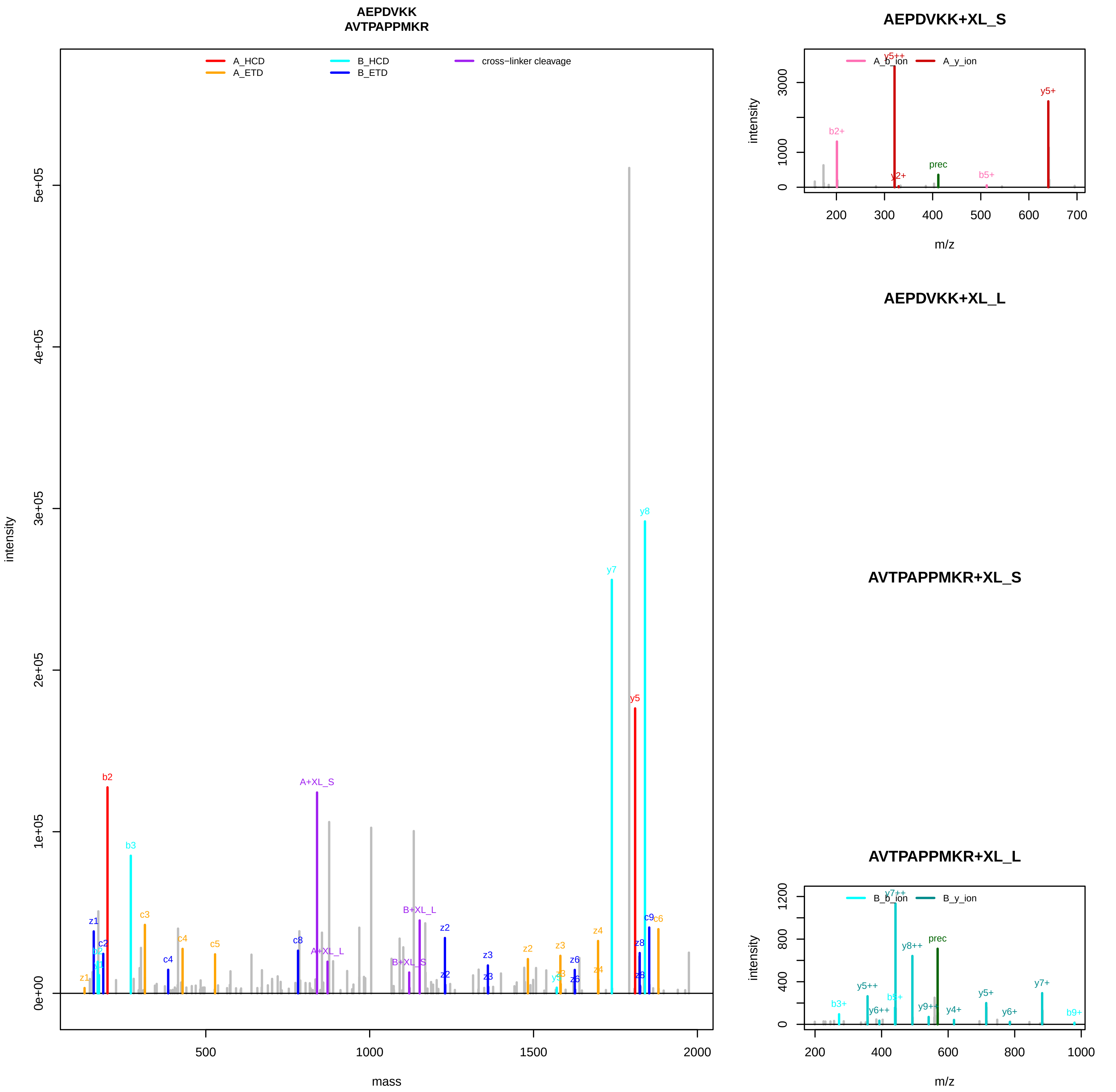
AEPDVKK
AVTPAPPMKR
A_HCD
A_ETD
B_HCD
B_ETD
cross−linker cleavage
intensity
mass
0e+00
1e+05
2e+05
3e+05
4e+05
5e+05
500
1000
1500
2000
b2
b3
c3
c4
c5
c8
A+XL_S
A+XL_L
B+XL_L
B+XL_S
z1
c2
z2
z3
z4
z6
z8
y5
y7
y8
c9
c6
AEPDVKK+XL_S
A_b_ion
A_y_ion
y5++
y5+
b2+
y2+
prec
b5+
m/z
AEPDVKK+XL_L
AVTPAPPMKR+XL_S
AVTPAPPMKR+XL_L
B_b_ion
B_y_ion
y7++
y8++
y5++
y6++
b3+
b5+
y9++
y4+
y5+
y6+
y7+
b9+
